# A Geometric Morphometrics Approach for Predicting Olfactory Region Accessibility: Toward Personalized Nose-to-Brain Drug Delivery

**DOI:** 10.3390/jpm15100461

**Published:** 2025-09-30

**Authors:** Priya Vishnumurthy, Thomas Radulesco, Gilles Bouchet, Alain Regard, Justin Michel

**Affiliations:** 1 Nemera La Verpillière, 69007 Lyon, France; 2 Aix Marseille University, CNRS, IUSTI, 13013 Marseille, France; 3 Aix Marseille University, APHM, CNRS, IUSTI, La Conception University Hospital, Oto-Rhino-Laryngology and Head and Neck Surgery Department, 13005 Marseille, France

**Keywords:** nose-to-brain, olfactory region accessibility, geometric morphometrics, semi-landmarks, nasal cavity variability, anatomical clusters, personalized medicine

## Abstract

**Background:** The anatomical variability of the nasal cavity affects intranasal drug delivery, especially to the olfactory region for nose-to-brain treatments. While previous studies used average models or 2D measurements to account for inter-individual variability, 3D shape variation of the region crossed by drug particles that target the olfactory area, namely the region of interest (ROI), remains unexplored to our knowledge. **Methods:** A geometric morphometric analysis was performed on the ROI of 151 unilateral nasal cavities from the CT scans of 78 patients. Ten fixed landmarks and 200 sliding semi-landmarks were digitized, using Viewbox 4.0, and standardized via Generalized Procrustes Analysis. Shape variability was analyzed through Principal Component Analysis. Morphological clusters were identified using Hierarchical Clustering on Principal Components, and characterized with MANOVA, ANOVA, and Tukey tests. **Results:** Validation tests confirmed the method’s reliability. Three morphological clusters were identified. Variations were significant in the X and Y axes, and minimal in Z. Cluster 1 had a broader anterior cavity with shallower turbinate onset, likely improving olfactory accessibility. Cluster 3 was narrower with deeper turbinates, potentially limiting olfactory accessibility. Cluster 2 was intermediate. Notably, 31.5% of patients had at least one cavity in cluster 1. **Conclusions:** Three distinct morphotypes of the region of the nasal cavity that potentially influence accessibility were identified. These findings will guide future computational fluid dynamics studies for optimizing nasal drug targeting and represent a practical step toward tailoring nose-to-brain drug delivery strategies in alignment with the principles of personalized medicine.

## 1. Introduction

The nasal cavity exhibits inter-individual variability that could be shaped by a combination of factors such as gender [1,2], age [3], ethnic origin [4,5], or climatic adaptation linked with different metabolic needs [6,7,8]. Such variability significantly impacts nasal airflow dynamics [9,10] and intranasal drug deposition patterns [11,12].

In recent years, intranasal drug administration has gained increasing interest, being a promising non-invasive route to deliver therapeutic agents directly to the central nervous system (CNS) through the olfactory nerves [13,14]. This route, namely the direct nose-to-brain pathway, bypasses the blood–brain barrier, which otherwise strongly limits the bioavailability of drugs for treating neurodegenerative diseases [15,16]. Optimizing drug delivery to the olfactory region is therefore of high relevance in the context of personalized medicine, where tailoring treatments to individual anatomical and physiological variability is essential to improve deposition efficiency.

However, due to the high inter-subject variability of the nasal cavity, a single nasal model is insufficient to accurately predict deposition outcomes for all individuals [17]. To address this issue, several studies have proposed using average anatomical models [18], sets of individual models [19], or selecting individuals based on different deposition efficiencies in specific zones [20,21]. Others have employed two-dimensional measurements of the nasal cavity to form subject groups and assess how specific anatomical parameters influence delivery [22]. While these approaches acknowledge the importance of anatomical variation, they do not directly investigate three-dimensional shape differences of the nasal cavity.

Geometric morphometrics is a mathematical and statistical method to quantitatively assess three-dimensional shape variation [23]. In this study, we applied a semi-landmark-based geometric morphometric approach [24] to assess the shape variability of the region that must be crossed by drug particles to reach the olfactory zone, namely the Region of Interest (ROI).

Our aim is to identify morphological variability associated with the accessibility of the olfactory region via clustering based on the 3D shape of the ROI, and to characterize these clusters using multivariate analysis. This approach could help design precise nose-to-brain drug delivery, paving the way for the development of stratified drug devices consistent with the principles of personalized medicine.

## 2. Materials and Methods

### 2.1. Ethical Considerations

This study was conducted according to the guidelines of the Declaration of Helsinki and approved by the AP-HM Institutional Review Board (N°2017-14-12-005) on 14 December 2017. All patients provided informed consent for the use of their data. Data were fully anonymized prior to analysis, ensuring that individual identification was not possible.

### 2.2. Study Sample

A total of 78 cranioencephalic computed tomography (CT) scans of patients admitted to the emergency room of a tertiary hospital for non-ENT diseases were collected. Patients had no known rhinologic history. CT scans were selected based on image quality and absence of major nasal pathologies. The study population comprised 42 females and 35 males (no demographic data were available for one adult patient), with a mean age of 53.9 years (range 15–85 years).

### 2.3. Surface Imaging and Pre-Processing

The 78 CT scans were imported into ITK-SNAP (version 3.8.0) in DICOM format, and a semi-automatic segmentation was performed to obtain 3D meshes of the nasal cavities. The thresholding mode, in which an intensity threshold was manually adjusted, was used to distinguish the nasal cavity lumen from the surrounding tissues. This procedure allowed the extraction of the nasal surface. The segmented volumes were exported in STL format. Paranasal sinuses were not considered for the segmentation, as they are not directly involved in the passage of therapeutic particles targeting the olfactory region.

Using the CAO tools of StarCCM+ (version 2310), each 3D nasal cavity mesh was cleaned, removing the segmentation artifacts, and separated into unilateral cavities. To ensure side-to-side comparability, the left nasal cavities were mirrored along the sagittal plane to be aligned with the right nasal cavities.

**Figure 1:**
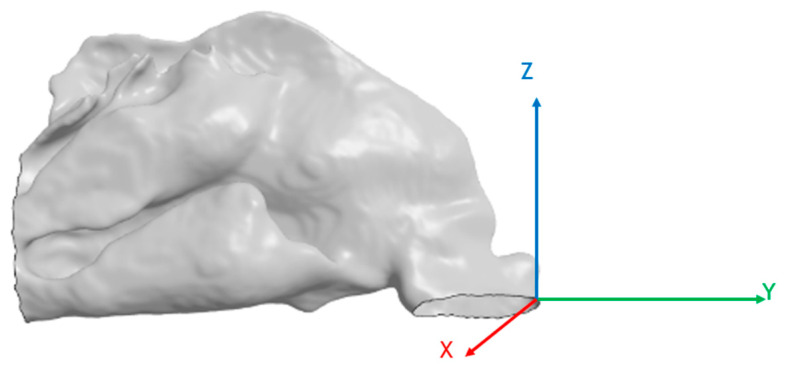
Origin and orientation of the nasal cavity meshes.

Out of the 78 patients, 5 had nasal probes in the right fossae. The right fossae of those patients were excluded from the study, leading to a total of 151 unilateral nasal cavities. The origin of each unilateral cavity was fixed at the angle between the nostril cutting plane and the front of the nasal cavity (Figure 1).

### 2.4. Definition of the ROI and Landmarks Digitization

The region of interest (ROI) was defined as starting from the plane crossing the plica nasi and the nasal valve, the narrower region of the nasal cavity, up to the anterior part of the olfactory region. The vestibule was excluded since it is primarily occupied by the delivery nozzle and does not influence particle trajectories within the nasal cavity. Beginning at the nasal valve ensures that only the functionally relevant intranasal passage is captured. Ending at the anterior olfactory region ensures that the zone of potential access to the olfactory mucosa is fully included.

**Figure 2:**
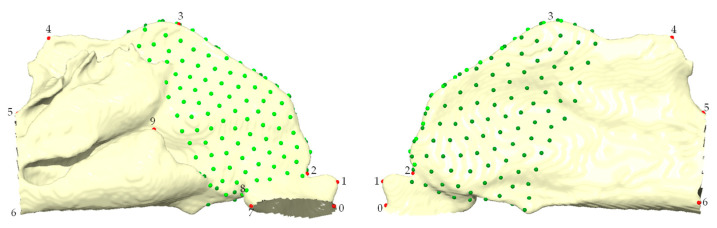
Three-dimensional template unilateral nasal cavity. Note: In red, the 10 fixed landmarks. In green, the 200 semi-landmarks. In light and dark green, the two patches analyzed.

Using Viewbox 4.0, a set of 10 fixed anatomical landmarks (Table 1) was first placed on a template unilateral nasal cavity model in homologous regions present in all the target patients. A total of 200 semi-landmarks were distributed across the ROI of the template model, organized into two patches to ensure optimal coverage (Figure 2). Semi-landmarks were projected from the template to each patient model using Thin Plate Spline (TPS) warping [25], using the method of bending energy minimization. This approach allowed semi-landmarks to slide tangentially along the surface, ensuring optimal homology across specimens while minimizing distortion [26].

All landmarking procedures were performed by the corresponding author. To further evaluate their reliability, intra- and inter-operator repeatability tests were conducted on a subset of models, as described in Section 2.8.

### 2.5. Shape Alignment and Principal Component Analysis (PCA)

All landmark coordinates were standardized via Generalized Procrustes Analysis (GPA) to remove variation due to translation, rotation, and scale. The aligned landmark coordinates were then analyzed using Principal Component Analysis (PCA) to identify the dominant axes of shape variation. The principal components (PCs) representing most of the variability were selected with the Elbow method. Analyses were conducted using the geomorph package in R (version 4.4.3) [27].

### 2.6. Cluster Identification

To classify morphological variations, Hierarchical Clustering on Principal Components (HCPC) was performed on the selected PCs. The analysis was conducted using the FactoMineR package in R (version 4.4.3) [27]. The number of clusters was determined automatically by the function (nb.clust = −1), which analyzes gains in cluster inertia to identify the partition that best reflects the underlying structure of the data. The optimal number of clusters has also been verified using the NbClust package in R (version 4.4.3) [27].

### 2.7. Cluster Characterization

Morphological differences between clusters were statistically evaluated by conducting statistical tests on the obtained clusters. A MANOVA test was conducted to identify the landmarks that are statistically different between at least two clusters on all the axes. An ANOVA test was conducted on each spatial coordinate to refine the results of the MANOVA test. A post-hoc Tukey’s test was performed on pairs of clusters to identify significant inter-cluster differences per landmark and axis.

### 2.8. Repeatability and Reproducibility Assessment

To assess landmark digitization reliability, a subset of fixed landmarks was manually placed twice by the same operator and once by a second operator on 20 models. Semi-landmarks were projected from the template as detailed in Section 2.4. Lin’s Concordance Correlation Coefficient (CCC) was used to quantify intra- and inter-operator agreement.

### 2.9. Bilateral Dimorphism Assessment

To test for potential bilateral asymmetry of shape, a Procrustes ANOVA test was conducted on the GPA-aligned coordinates of the left and right nasal cavities. To assess potential bias due to the unequal number of left and right cavities, Procrustes ANOVA was repeated after excluding the five left cavities corresponding to patients with probes in the right cavity.

### 2.10. Optimal Sample Size

To assess the sample size sufficiency for PCA stability, a resampling analysis was performed. PCA was applied on randomly selected subsets of increasing size (*n* = 20 to 150), repeated 100 times per sample size.

## 3. Results

CCC values indicated good to excellent repeatability and reproducibility for all fixed and semi-landmarks (Table 2). The Procrustes ANOVA test indicated no significant lateral dimorphism (*p* = 0.844) (Appendix A.1). Sensitivity analysis of the Procrustes ANOVA revealed no bias from the five left cavities corresponding to patients with probes in the right cavity (*p* = 0.957) (Appendix A.2). Consequently, the right and left cavities were combined for the remaining PCA and clustering analyses. The first five principal components (PCs) were retained for the clustering analysis based on the elbow method, which captured approximately 60% of total shape variance (Appendix B). The variance explained by the first five PCs stabilized around 50 unilateral cavities (Appendix C), confirming that the sample of 151 unilateral cavities was sufficient to ensure robust and stable PCA outcomes.

**Figure 3:**
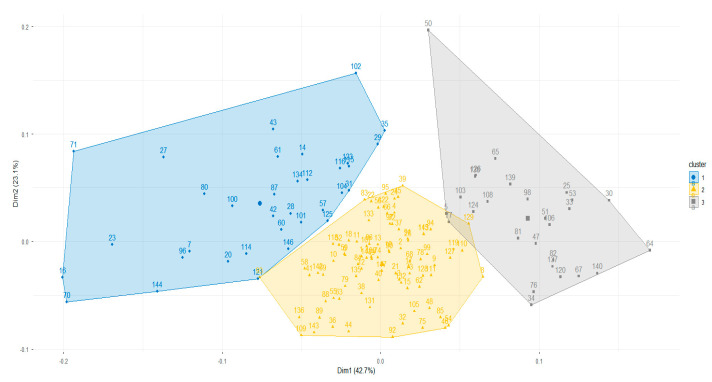
Factor map from the HCPC analysis. Individuals are represented in the first two principal components (Dim1 and Dim2), grouped into three clusters (blue, yellow, and grey).

Three clusters were identified by HCPC based on inertia gain (Figure 3), in agreement with the majority of 30 clustering validity indices (Appendix D). Clusters 1, 2, and 3 were composed of 34, 89, and 28 unilateral cavities, respectively. The demographic characteristics of the clusters are presented in Table 3. Kruskal–Wallis and Chi-square tests revealed no significant differences between demographic characteristics and the obtained clusters.

The MANOVA test revealed that four landmarks over the 200 did not significantly differ between the three clusters over the three axes. The ANOVA test revealed limited significant differences between the three clusters regarding the Z axis, but revealed regions that differ significantly on the axes X and Y. The pairwise comparison of the clusters conducted with the Tukey test revealed regions significantly different between the two clusters on each axis (Table 4). Regarding the posterior-superior part of the ROI, on the X axis, cluster 1 is wider than clusters 2 and 3, which do not differ in this region. On the Y axis, cluster 3 is the largest, cluster 1 the smallest, and cluster 2 is intermediate. Regarding the anterior-superior part of the ROI, on the X axis, cluster 1 is the widest, cluster 3 is the narrowest, and cluster 2 is intermediate. On the Y axis, cluster 1 has a less convex frontal edge than clusters 2 and 3, which do not differ in this region. Regarding the anterior-superior part, on the X axis, cluster 3 is wider than clusters 1 and 2, which do not differ in this area. On the Y axis, cluster 1 is shallower than clusters 2 and 3, which do not differ in this area. Regarding the anterior-inferior part, on the X axis, cluster 2 is wider than clusters 1 and 3, which do not differ in this zone. On the Y axis, cluster 3 has the most rounded frontal edge, cluster 1 the least rounded, and cluster 2 is intermediate.

The right and left sides of the same patient were associated, and the pair of clusters formed, independently of the laterality, was quantified in Table 5. The five patients, for whom only one left side was analyzed, were excluded from this analysis. Of the 73 patients, 31.5% have at least one cavity pertaining to cluster 1; 69.9% have at least one cavity pertaining to cluster 2; and 26% have at least one cavity pertaining to cluster 3.

**Figure 4:**
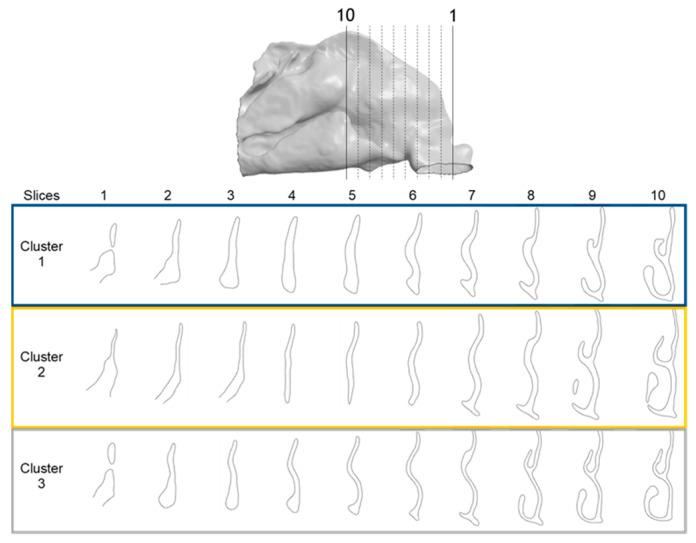
Coronal sections of the ROI of the representative patients of each cluster, numbered from 1 (anterior) to 10 (posterior), as shown in the 3D nasal cavity model.

The representative patients of each cluster have been selected, and the ROI has been sliced into ten parts to see the significant difference between the three clusters (Figure 4).

## 4. Discussion

### 4.1. Summary of Key Findings

This study used a geometric morphometric approach to assess the morphological variability of the anterior region of the nasal cavity, a critical zone to access the olfactory region and, therefore, for efficient nose-to-brain drug delivery. Three distinct morphological clusters were identified based on shape characteristics.

Among these clusters, cluster 1 displayed the widest superior regions with a shallower ROI, a morphology that may favor accessibility to the olfactory region from the nostril. In contrast, cluster 3 was characterized by a narrower and deeper profile, with an increased curvature of the frontal edge, features that may create anatomical barriers to efficient transport to the olfactory region. Cluster 2 showed intermediate characteristics, with a broader anterior-inferior region compared with the other two clusters. The predictions for the olfactory accessibility of cluster 2 were more complex.

By identifying anatomies that are favorable for olfactory deposition, the findings highlight that the morphological variability of the anterior nasal cavity may have major implications for nose-to-brain drug delivery. Previous studies have demonstrated that the nasal cavity geometry modulates particle trajectories and deposition efficiency [10,11,17]. However, most of these studies rely on either single-subject models or population averages, which may hide critical shape-driven inter-individual variability. In contrast, our use of semi-landmarks and a shape-based clustering method provides a more detailed characterization of morphological diversity in a clinically relevant region.

### 4.2. Methodological Considerations and Limitations

From a methodological perspective, we demonstrated good reproducibility and repeatability of landmark digitization, confirmed the absence of significant lateral asymmetry, verified that our sample size was sufficient to ensure stable statistical analyses, and validated the optimal number of clusters for our dataset. These validation results increase confidence in the strength of the observed morphological patterns.

Nonetheless, semi-landmarks were projected from a single template using TPS warping. Relying on a single template may introduce template bias, since the configuration of the chosen specimen can influence how semi-landmarks are distributed across all individuals [28,29]. Several strategies have been proposed to reduce this limitation, including the use of multiple templates [30] or projection onto a mean shape [31]. In the present study, the template mesh was selected to avoid severe septal deviation or stenosis, ensuring a neutral and representative model. Moreover, the iterative minimization of bending energy helps to limit distortions by allowing semi-landmarks to slide tangentially along the surface, which partially mitigates but does not completely remove the risk of template bias. Future studies could therefore compare results obtained from different templates or consensus shapes to ensure robust morphotype identification.

Regarding the study population, neither age nor sex showed any correlation with the obtained clusters, suggesting that these demographic characteristics may not impact the accessibility of the olfactory region of an adult population. However, our dataset only included non-pediatric individuals, which restricted the generalizability of these findings. Developmental and aging processes also need to be considered; the nasal skeleton and mucosa undergo marked changes from childhood to adolescence, while remodeling and atrophy can occur later in life [3]. Moreover, the patients considered in this study had no known rhinologic history, whereas dynamic physiological or pathological conditions, such as inflammatory episodes, can temporarily alter the functional lumen of the nasal cavity [32,33], which restricts the scope of clinical applicability of our morphotypes. Ethnic origin is also known to modulate nasal cavity geometry and modulate airflow and deposition patterns. These dimensions were not represented in our sample and warrant dedicated investigation in future studies using a broader framework that integrates structural, functional, and patient-specific information to refine patient stratification and strengthen the translational potential of morphotype-based approaches.

Finally, although the clustering was data-driven and based on shape, the precise mechanisms by which these morphologies influence the accessibility of the olfactory region remain speculative without direct CFD simulations.

### 4.3. Clinical Implications and Perspectives

Fully individualized devices tailored to each patient’s anatomy are unlikely to be feasible in clinical practice due to their cost and complexity. Instead, our results support a morphotype-based strategy, in which a limited set of representative anatomical configurations could guide the development of devices optimized for each profile. This approach offers a compromise between full personalization and universality; it allows stratification into a few clinically relevant subtypes, while allowing device manufacturers to develop a small set of optimized geometries or spray nozzles that collectively cover most patients.

Computational fluid dynamics (CFD) simulations applied to these morphotypes will be essential to identify which design parameters (spray angle, plume geometry, particle size distribution) are most robust across clusters, and which may require specific adaptations for less favorable morphologies, such as cluster 3. In this way, the morphotypes described here can serve as a translational bridge between anatomical variability and the design of practical, effective, and clinically deployable nose-to-brain drug delivery devices.

## 5. Conclusions

This study identifies distinct morphological patterns in the region of the nasal cavity that potentially influence accessibility to the olfactory area. Future CFD simulations will be essential to validate the proposed association between shape and accessibility. By establishing anatomical clusters that can guide device optimization and patient stratification, our work proposes a practical pathway to tailor nose-to-brain drug delivery devices in line with the principles of personalized medicine.

## Figures and Tables

**Table 1:** Descriptions of the fixed landmarks.

Landmarks	Definition
0	Most anterior maximum at the angle between the nostril cutting plane and the front of the nasal cavity.
1	Most anterior maximum of the vestibule.
2	Highest point of the nasal valve, corresponding to the narrowest superior point between the vestibule and the nasal fossa.
3	Highest point of the nasal cavity, which is located at the front of the olfactory region.
4	Highest point of the nasal cavity, which is located at the back of the olfactory region.
5	Highest point of the choana, which is not aligned with the extension of the turbinate.
6	Lowest point of the nasal cavity, which is positioned closest to the nasal septum.
7	Most posterior maximum on the nostril cutting plane
8	Narrowest inferior point of the nasal valve
9	Highest anterior point of the inferior meatus

**Table 2:** Lin’s CCC for repeatability and reproducibility assessment.

Landmarks	Repeatability	Reproducibility
Fixed	0.934	0.877
Sliding	0.992	0.977

**Table 3:** Demographic characteristics and associated *p*-values of the obtained clusters. Note: No data on sex and age were available for one patient who had a right cavity in cluster 2 and a left cavity in cluster 3. ^a^ *p*-value obtained with a Chi-square test; ^b^ *p*-value obtained with a Kruskal–Wallis test.

Variable	Cluster 1	Cluster 2	Cluster 3	*p*-Value
Number of unilateral cavities (N (%))	34 (22.5)	89 (58.9)	28 (18.5)	-
Side (Right:Left)	17:17	44:45	12:16	0.812 ^a^
Sex (Female:Male)	15:19	47:41	19:8	0.119 ^a^
Age (Mean ± standard deviation)	51.7 ± 20.4	54.5 ± 20.1	53.2 ± 17.4	0.678 ^b^

**Table 4:** Results of the pairwise analysis of the clusters using the Tukey test represented on the nasal cavities. Note: In black, landmarks with no significant difference; In red or blue, landmarks with significant differences, in red, landmarks in cluster A are further from the fixed origin shown in Figure 1 than cluster B, in blue, landmarks in cluster B are further from the origin than cluster A.

Axe	A = Cluster 2 B = Cluster 1	A = Cluster 3 B = Cluster 1	A = Cluster 3 B = Cluster 2
X	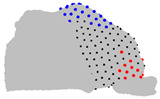	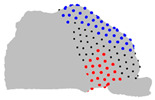	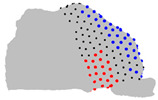
	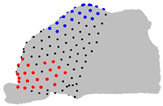	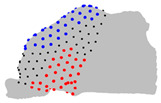	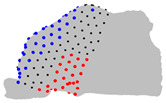
Y	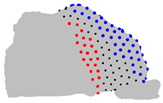	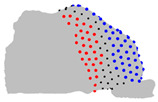	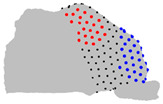
	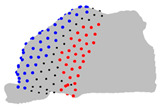	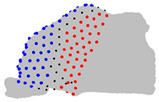	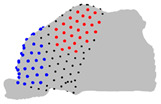
Z	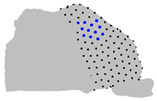	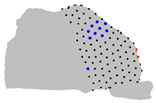	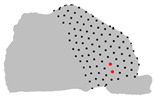
	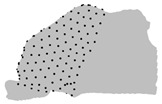	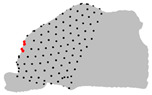	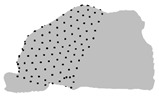

**Table 5:** Quantification of the pairs of clusters in patients.

Pair of Clusters	Number of Patients	% of Total Patients
1-1	11	15.1%
1-2	10	13.7%
1-3	2	2.7%
2-2	33	45.2%
2-3	8	11.0%
3-3	9	12.3%

**Figure A1:**
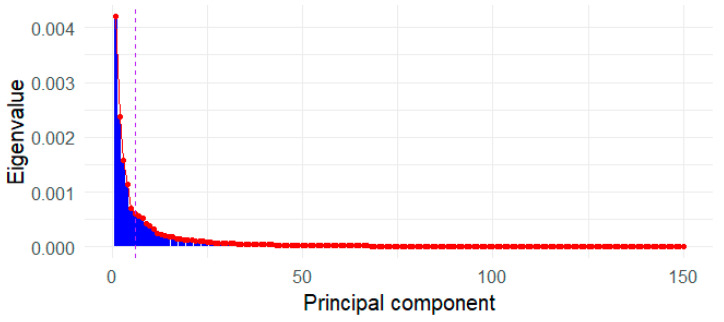
Selection of the PCs to include in the HCPC analysis with the Elbow method. Note: In red points, the eigenvalues; in blue bars the eigenvalue distribution; in dashed purple line, the location of the Elbow.

**Figure A2:**
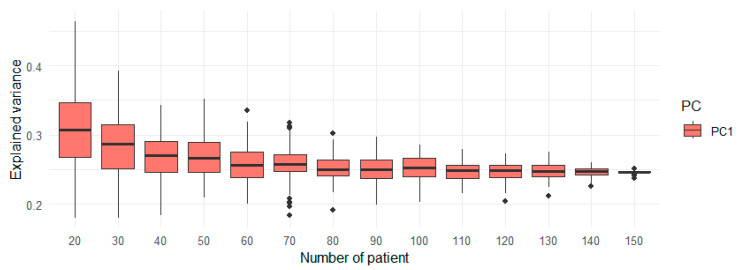
Boxplot of the explained variance as a function of the number of patients.

## Data Availability

Statistical data will be available upon reasonable request.

## Appendix A. Bilateral Dimorphism Assessment

### Appendix A.1. Procrustes ANOVA Test on 151 Unilateral Nasal Cavities

**Table A1:** Procrustes ANOVA to assess for bilateral dimorphism among the 151 unilateral cavities.

	Df	SS	MS	Rsq	F	Z	p
Laterality	1	0.01061	0.010613	0.00417	0.6241	−1.0364	0.844
Residuals	149	2.53374	0.017005	0.99583			
Total	150	2.54435					

### Appendix A.2. Sensitivity Analysis Excluding Left Cavities of the Patients with Probe in the Right Side

**Table A2:** Procrustes ANOVA to assess for bilateral dimorphism among the 145 unilateral cavities of the patients with no probe in the right cavity.

	Df	SS	MS	Rsq	F	Z	p
Laterality	1	0.0086952	0.0035	0.506	0.0087	−1.6843	0.957
Residuals	144	2.4746	0.0171848	0.9965			
Total	145	2.4833					

## Appendix D. Validation of the Optimal Number of Clusters

**Table A3:** Clustering validity indices and suggested optimal number of clusters.

Index	Optimal Number of Clusters Suggested	Index Value
KL	5	17.7189
CH	2	43.3387
Hartigan	15	9.1558
CCC	15	−2.8463
Scott	3	117.9024
Marriot	4	0.0002
TrCovW	3	0.0207
TraceW	3	0.0768
Friedman	4	2.0838
Rubin	5	−0.0796
Cindex	14	0.219
DB	15	1.3075
Silhouette	3	0.2452
Duda	2	1.4595
PseudoT2	2	−38.0942
Beale	2	−0.9695
Ratkowsky	5	0.2303
Ball	3	0.2651
PtBiserial	3	0.4999
Frey	1	NA
McClain	2	0.758
Dunn	15	0.1148
Hubert	0	0
SDindex	8	52.2909
Dindex	0	0
SDbw	14	0.3151
Gap	2	0.0153
Gamma	15	0.79
Gplus	15	84.5262
Tau	3	1769.315
